# Cornual Pregnancy: Results of a Single-Center Retrospective Experience and Systematic Review on Reproductive Outcomes

**DOI:** 10.3390/medicina60010186

**Published:** 2024-01-21

**Authors:** Fathi Mraihi, Giovanni Buzzaccarini, Antonio D’Amato, Antonio Simone Laganà, Jihene Basly, Chaima Mejri, Montasar Hafsi, Dalenda Chelli, Zaineb Ghali, Bianca Bianco, Fabio Barra, Andrea Etrusco

**Affiliations:** 1 Gynecology and Obstetrics Unit “D”, Maternity and Neonatology Center, Tunis University Hospital, Tunis 1001, Tunisia; mraihi.fethi@gmail.com (F.M.); basly.jihene@gmail.com (J.B.); chaymamejri45@gmail.com (C.M.); montahafsi17@gmail.com (M.H.); dalenda.chelli@sted-tn.com (D.C.); ghalizeineb6@gmail.com (Z.G.); 2 Department of Obstetrics and Gynecology, IRCCS San Raffaele Scientific Institute, Vita-Salute San Raffaele University, 20132 Milan, Italy; giovanni.buzzaccarini@gmail.com; 3 1st Unit of Obstetrics and Gynecology, Department of Interdisciplinary Medicine, University of Bari, 70124 Bari, Italy; antoniodamato19@libero.it; 4 Unit of Obstetrics and Gynecology, “Paolo Giaccone” Hospital, Department of Health Promotion, Mother and Child Care, Internal Medicine and Medical Specialties (PROMISE), University of Palermo, 90127 Palermo, Italy; etruscoandrea@gmail.com; 5 Discipline of Sexual and Reproductive Health and Populational Genetics, Department of Collective Health, Faculdade de Medicina do ABC/Centro Universitário FMABC, Santo André 09060-870, SP, Brazil; bianca.bianco@einstein.br; 6 Unit of Obstetrics and Gynecology, P.O. “Ospedale del Tigullio”-ASL4, Via G. B. Ghio 9, Metropolitan Area of Genoa, 16043 Chiavari, Italy; fabio.barra@icloud.com; 7 Department of Health Sciences (DISSAL), University of Genoa, Via Antonio Pastore 1, 16132 Genova, Italy

**Keywords:** cornual pregnancy, ectopic pregnancy, laparoscopy, methotrexate, fertility

## Abstract

*Background and Objectives*: Cornual pregnancies (CPs) are rare forms of ectopic pregnancy. When abortion does not occur, it can be a life-threatening condition for the mother and can also impair future fertility. We present our experience in the diagnosis and management of CPs. A systematic review was also conducted to investigate the reproductive outcomes after treatment. *Materials and Methods*: Between January 2010 and December 2022, we performed a retrospective, cross-sectional, single-center, and descriptive data collection and analysis (ClinicalTrial ID: NCT06165770). The search for suitable articles published in English was carried out using the following databases (PROSPERO ID: CRD42023484909): MEDLINE, EMBASE, Global Health, The Cochrane Library (Cochrane Database of Systematic Reviews, Cochrane Central Register of Controlled Trials, and Cochrane Methodology Register), Health Technology Assessment Database, Web of Science, and search register such as ClinicalTrial. Only studies describing the impact of CP treatment on fertility were selected. *Results*: Two studies were included in the systematic review. Seventeen patients suffering from CPs were selected. In our series, a pelvic ultrasound allowed for the diagnosis of a cornual localization in 35.30% of cases. Thirteen women (76.47%) underwent immediate surgical management. The laparoscopic approach was the most used (76.92%), with a laparotomic conversion rate of 30%. Four patients (23.52%) received medical treatment with methotrexate. After treatment, two patients managed to achieve pregnancy. *Conclusions*: CP is a rare form of ectopic pregnancy that can quickly become life-threatening for the mother. Ultrasound does not lead to a precise diagnosis in all cases. In the absence of complications and emergencies, laparoscopy is an approach that could be considered valid. For selected asymptomatic patients, medical treatment may be a valid alternative. The data from the studies included in the systematic review, although demonstrating a superiority of medical treatment in terms of future pregnancies, are heterogeneous and do not allow us to reach a definitive conclusion.

## 1. Introduction

Cornual pregnancies (CPs) are rare forms of ectopic pregnancy (EP) and cumulatively account for up to 2% of all EPs [1]. CP is defined as a pregnancy in which the implantation and development of the product of conception occurs in the upper and lateral portions of the uterus at the level of the tubal recesses. Sometimes it can be mistakenly confused with an interstitial pregnancy (IP) with evolution to the uterine cavity. Up to 3/4 of these pregnancies end in miscarriage around the 12th week of pregnancy; when this does not occur, it can be a life-threatening condition for the mother [2]. Because uterine angles are characterized by a particularly thin myometrium, uterine rupture can occur even in the absence of excessive dilation exerted by the gestational sac. On vaginal examination, the uterus appears conspicuously deformed at one (or both, in the case of bilateral cornual pregnancies [3]) of the tubal angles. Complete aspiration of the product of conception is extremely difficult, and the possible need to resort to endouterine instrumental maneuvers can be particularly dangerous, as it is fraught with high risks of uterine hemorrhage and/or perforation [4]. Today, nontubal pregnancies, particularly CPs, represent a major diagnostic and therapeutic challenge, mainly because of their low prevalence, the need for ultrasound expertise to make the diagnosis, and the lack of specific recommendations for management. Conventionally, treatment of this condition can make use of medical or surgical treatment. The various treatment options depend on the pregnant woman’s age and medical history, the size of the gestational sac, the β-human chorionic gonadotropin (hCG) level, the presence of embryonic heartbeat, and the presence of any urgent conditions, such as the presence of hemoperitoneum. In the specific case of CPs, a wait-and-see approach is difficult to apply and requires a risk–benefit assessment. For medical therapy, methotrexate (MTX) is mainly used [5]. In hemodynamically unstable patients with signs and/or symptoms of acute abdomen, hemoperitoneum, and/or gestational sac diameter greater than 3.5 cm, medical therapy is contraindicated, and surgical treatment should be used [5,6]. The surgical approach may range from uterine horn resection to hysterectomy in urgent and more complex cases. Although the laparotomic approach is well described and widely used in urgent and complicated cases of CP, the literature is rich with evidence on the conservative and minimally invasive approach provided by laparoscopy [7,8,9,10,11]. This study was thus developed to review our single-center experience in the management of CP, evaluating the diagnostic path and medical and surgical management, as well as any complications, of this rare form of EP. A systematic review was also performed to evaluate the impact of treatment decisions on subsequent fertility prognosis.

## 2. Materials and Methods

### 2.1. Study Design and Data Collection

We conducted a retrospective, cross-sectional, single-center, descriptive study (ClinicalTrial ID: NCT06165770) collecting cases of CP at the Gynecology and Obstetrics Unit “D” of Tunis University Hospital, Maternity and Neonatology Center. Patient data were collected between January 2010 and December 2022. Considering the retrospective nature of the study and consistency with the REporting of studies Conducted using Observational Routinely collected Health Data (RECORD) statement, with no experimental intervention by the investigators and with anonymized data collection, a formal ethics committee approval was waived. All the patients were fully informed and signed informed consent for all the procedures as well as data collection and analysis for research purposes. No remuneration was offered to give consent to be enrolled in the study. The study was not advertised.

The inclusion criteria were as follows: age ≥ 18 years and patients with a diagnosis of CP. All patients whose medical records did not contain the data deemed necessary for study analysis or who did not consent to the publication and use of their data were excluded from the study. We used a standardized, computerized data collection form with several sections (general information, clinical investigations, complementary investigations, and treatment). We recorded age, medical, surgical, and gynecological and obstetric history, duration of amenorrhea, date of hospitalization, and possible risk factors for ectopic pregnancy. Clinical examination, pelvic ultrasound data, medical and surgical treatments, intra- and postoperative complications, length of hospital stay, and the effect of treatment on subsequent fertility were also recorded in detail.

### 2.2. Medical and Surgical Treatment

According to the intervention protocol adopted by our study, medical treatment was reserved for women with an uninterrupted, anamnestically dated CP of less than 8 weeks of amenorrhea, with a β-hCG level not exceeding 10,000 IU/L. All patients undergoing medical treatment received an appropriate explanation of the procedures, and treatment was performed once signed informed consent was obtained. As indicated by leading scientific societies [5,6], we used MTX as medical therapy. MTX is an anticancer drug belonging to the class of antimetabolites. By inhibiting the enzyme dihydrofolate reductase (DHFR), it is a folic acid antagonist and thus interferes with DNA synthesis and cell growth. MTX also has anti-inflammatory and immunosuppressive properties, making it also indicated for the treatment of conditions other than neoplastic diseases; it has also been shown to produce long-term remission of human trophoblastic tumors. It is readily absorbed regardless of the route of administration, binds at about 35% to plasma proteins, and disappears from plasma in a triphasic manner. The rapid distribution phase is followed by a second phase with a half-life of about 2 h and a final phase with a half-life of about 8 h. The affinity of MTX for DHFR is about 1000 times that of folate for DHFR. DHFR catalyzes the conversion of dihydrofolate to active tetrahydrofolate. MTX also directly inhibits the enzyme thymidylate synthase, as well as interfering with folate-dependent enzymes involved in the metabolic pathway of purine neosynthesis. In this way, the drug interferes with both the synthesis and repair of cellular DNA and the replication of the cell itself. The cytotoxic action of the molecule is closely linked to the cell cycle and occurs specifically during RNA and DNA synthesis, i.e. during the S phase of the cell cycle. Logically, therefore, the drug has a greater toxic effect on rapidly multiplying tissues and on cells with a high division rate and high growth fraction. Actively proliferating tissues (such as malignant cells or embryonic cells), which are characterized by more frequent replication of their DNA, are normally more sensitive to the effects of the molecule. Our medical treatment consisted of the injection of 1 mg/kg MTX intracameral injection combined with an intramuscular injection of the same drug at the same dosage. Two techniques were used to inject MTX inside the gestational sac: ultrasound-guided injection and laparoscopic-assisted injection. The choice between the two techniques was related to the possibility of accurate diagnosis of the location of EP and the risk of hemorrhage. A second intramuscular injection of MTX 1 mg/kg was administered to all patients who had persistently elevated β-hCG levels at follow-up on day 4 after the first administration. Medical treatment success was defined as a greater than 15% reduction in β-hCG levels from baseline on day 7 after injection. The choice of the laparotomic (L/T) versus laparoscopic (L/S) approach was based on the presence or absence of hemoperitoneum and/or hemodynamic instability. Surgical treatment consisted of fertility-sparing treatment with resection of the uterine horn and ipsilateral fallopian tube. In the presence of hemoperitoneum, the latter was drained. Conversion of L/S to L/T occurred only in urgent cases, which were deemed essential to safeguard the patient’s life. In patients undergoing surgical treatment, if elevated β-hCG levels persisted, intramuscular injection of 1 mg/kg MTX was administered.

### 2.3. Data Analysis

Data were analyzed using SPSS version 25.0 statistical software. Qualitative variables were described as absolute frequencies and percentages. Descriptive statistics were reported according to the data distribution as the mean ± SD when they had a Gaussian distribution; the categorical variables were reported as absolute number and percentage (%).

### 2.4. Eligibility Criteria for the Systematic Review

Only original studies (retrospective or prospective) reporting CP treatment and its impact on fertility were considered eligible for inclusion in this systematic review. The impact on fertility was defined as seeking pregnancy after undergoing treatment for CP. No restriction on treatment modality was applied. Studies that described only the technique of the procedure (step-by-step description of the procedure) were excluded.

### 2.5. Information Sources

This systematic review (PROSPERO ID: CRD42023484909) was carried out according to the Preferred Reporting Items for Systematic Reviews and Meta-Analyses (PRISMA) guidelines [12], available through the Enhancing the Quality and Transparency of Health Research (EQUATOR) network, and the Cochrane Handbook for Systematic Reviews [13]. MEDLINE, EMBASE, Global Health, The Cochrane Library (Cochrane Database of Systematic Reviews, Cochrane Central Register of Controlled Trials, Cochrane Methodology Register), Health Technology Assessment Database, Web of Science, and Research Register (such as ClinicalTrial) were searched for studies describing the impact of the CP treatment on fertility.

### 2.6. Search Strategy

The following medical subject heading (MeSH) and key search terms were used: “Cornual pregnancy” (MeSH Unique ID: D065173) AND “Fertility” (MeSH Unique ID: D005298) OR “Infertility” (MeSH Unique ID: D007246) OR “Methotrexate” (MeSH Unique ID: D008727) OR “Laparoscopy” (MeSH Unique ID: D010535) OR “Conservative treatment” (MeSH Unique ID: D000072700) OR “Uterine horn resection” OR “Cornuostomy” OR “Salpingectomy” (MeSH Unique ID: D058994). Given the rarity of this clinical condition, non-English-language studies that met the inclusion criteria were also considered eligible. We selected papers from the inception of each database until 30 September 2023.

### 2.7. Study Selection

Titles and/or abstracts of studies retrieved using the search strategy were screened independently by 2 review authors (A.E. and A.S.L.) to identify studies that met the inclusion criteria. The full texts of these potentially eligible articles were retrieved and independently assessed for eligibility by two other review team members (G.B. and A.D.A.). A manual search of the references of the included studies was conducted to prevent the omission of pertinent research. Any disagreement between them over the eligibility of articles was resolved through discussion with a third (external) collaborator. All authors approved the final selection.

### 2.8. Assessment of Risk of Bias

Two reviewers (A.E. and A.S.L.) independently assessed the risk of bias in the studies included in this systematic review using a modified version of the Newcastle–Ottawa Scale (NOS) [14]. The quality of the studies was evaluated in the following five different domains: “study design and sample representativeness”, “sampling technique”, “description of the medical and/or surgical technique”, “quality of the population description”, and “incomplete outcome data” (Table S1). Any disagreements between the reviewers were resolved by a third reviewer (F.B.).

### 2.9. Data Extraction

Two authors (A.E. and A.S.L.) independently extracted data from articles about study features, characteristics of the included populations, medical and surgical procedures, complications, and results/outcomes using a prepiloted standard form to ensure consistency. One author (B.B.) reviewed the entire data extraction process.

### 2.10. Outcomes

The following outcomes were measured: the clinical pregnancy rate (CPR; defined as the presence of a gestational sac on transvaginal ultrasound or other definitive clinical signs); the ongoing pregnancy rate (OPR; defined as a pregnancy beyond 24 weeks of gestation); the miscarriage rate (MR; defined as fetal loss before the 20th week of gestation).

## 3. Results

### 3.1. Single-Center Experience

Between January 2010 and December 2022, based on inclusion and exclusion criteria, 17 women affected by CP came to the Gynecology and Obstetrics Unit “D” of Tunis University Hospital, Maternity and Neonatology Center. The mean study population age was 36 ± 4.45 (range 24–41) years. All of them were multiparous; the mean number of previous pregnancies was 4 ± 2.04 and the mean number of previous miscarriages was 1 ± 1.05. Only one (5.88%) of the recruited patients achieved pregnancy with assisted reproductive technology (ART). A thorough analysis was made of the presence of possible risk factors that could cause EP. None of the recruited patients had a previous history of EP or pelvic inflammatory disease (PID). None of the patients had congenital abnormalities of the genital system. One patient (5.88%) suffered from infertility, and therefore achieved pregnancy via ART. None of the patients had previously undergone abdominopelvic surgery; however, a mean of 1.05 ± 0.74 uterine cavity revisions due to previous abortions was recorded. One patient (5.88%) had a copper intrauterine device (IUD) at the time of conception; two patients (11.76%) were taking oral contraceptives. None of the patients smoked. The complete baseline characteristics of the patients are shown in Table 1. The gestation period in which the patients came to our attention was a mean of 6.85 ± 1.55 weeks of amenorrhea. The two most common reasons for consultation were pelvic pain (n = 15; 88.23%) and metrorrhagia (n = 10; 58.82%). In contrast, seven patients (41.18%) reported amenorrhea; speculum examination of these patients also revealed the presence of a minimal dark blood discharge of endouterine origin. On palpation, four patients (23.53%) had a deformed uterus at either angle. Three patients (17.64%) presented with hemorrhagic shock and hemoperitoneum. The complete list of signs and symptoms is shown in Table 1. The mean β-hCG level was 15,708.06 ± 7042.06 UI/L. After an initial assessment, all patients underwent preliminary US evaluation. The US diagnosis of CP was defined by the presence of an empty uterine cavity associated with an eccentric gestational sac asymmetrically surrounded by endometrium. However, although all patients had an empty uterine cavity, only six patients (35.30%) had a US diagnosis. In addition, in three patients (17.65%), hemoperitoneum was evident on US.

In our series, four patients (23.53%) had a gestational age less than 8 weeks of amenorrhea and β-hCG values less than 10,000 IU/L, and therefore received medical treatment. For one (25%) of these patients, we were able to make an US diagnosis of CP, and she underwent a US-guided injection of 1 mg/kg MTX in situ combined with an intramuscular injection of 1 mg/kg MTX. In contrast, for the remaining three patients (75%), the localization of the pregnancy could not be defined by US, and because the hemorrhagic risk was high, they underwent an L/S-assisted injection of 1 mg/kg MTX in situ combined with an intramuscular injection of 1 mg/kg MTX. The diagnosis of CP in these patients was performed intraoperatively and was defined by the presence of normal, nondistended fallopian tubes, and the presence of a distended uterine horn with thinned myometrium. For two patients (50%) who underwent medical treatment (both in the L/S-assisted injection group), a second 1 mg/kg intramuscular injection of MTX was needed because the β-hCG levels were still elevated on day 4 after the procedure. The overall mean time to β-hCG negativization in medically treated patients was 36.4 days. The reported efficacy was 100%. The remaining patients (n = 13; 76.47%) underwent immediate surgical treatment. All patients underwent uterine horn resection combined with unilateral salpingectomy. The L/S approach was the most frequently used (n = 10; 76.92%). The recorded L/T conversion rate was 30%. Conversion to L/T was necessary in case of significant blood loss. Only two (20%) patients undergoing L/S developed severe postoperative anemia that was resolved by transfusion. Since three patients (17.65%) presented with hemoperitoneum associated with hemorrhagic shock, direct L/T (23.08%) was chosen. None of these patients developed intraoperative complications, and one of them (33.33%) developed postoperative ileus, which was resolved with fasting, nasogastric tube placement, and hydration. Only three (23.08%) of the patients who underwent surgical treatment needed a second procedure (intramuscular injection of 1 mg/kg MTX). Complete data about diagnosis and treatment are shown in Table 2.

The definitive histological examination confirmed the criteria for the diagnosis of CP in all the cases included in this study: the presence of chorionic villi at the level of the uterine horn without invasion of the tubal isthmus, thus allowing a definite diagnosis of CP and strictly distinguishing it from IP. In our series, only seven women (41.18%) expressed willingness to seek a new pregnancy after the CP treatment. Of these, one (50%) underwent a US-guided injection of 1 mg/kg MTX in situ combined with an intramuscular injection of 1 mg/kg MTX, and achieved one natural pregnancy; one (50%) underwent an L/S-assisted injection of 1 mg/kg MTX in situ combined with an intramuscular injection of 1 mg/kg MTX and achieved one pregnancy by ART. No spontaneous abortions were recorded. Complete data concerning fertility after treatment for CP are shown in Table 3.

### 3.2. Study Selection

**Figure 1:**
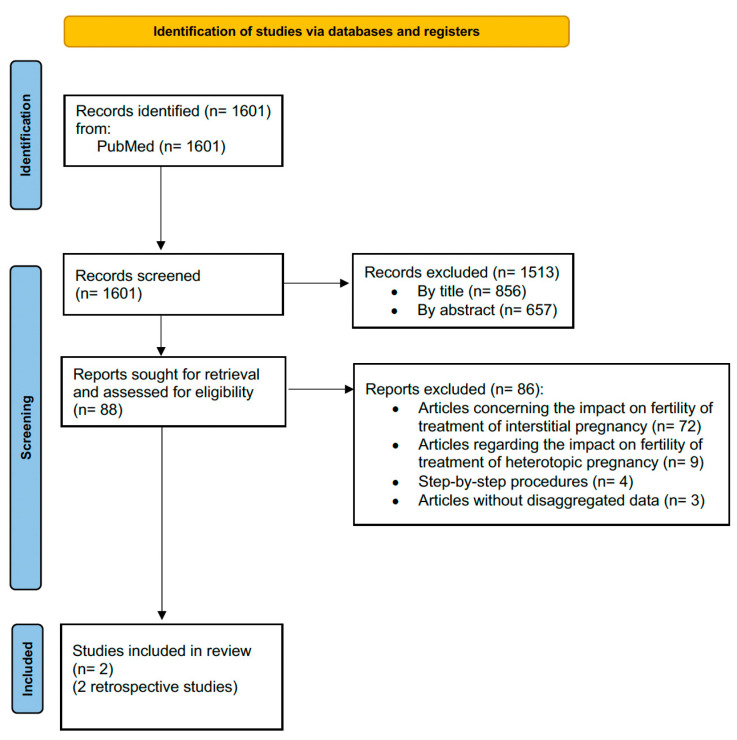
Preferred Reporting Items for Systematic Reviews and Meta-Analyses (PRISMA) flow diagram.

Study selection for the systematic review is displayed in Figure 1. After the evaluation of full texts, a total of two papers [15,16] that met the abovementioned inclusion criteria were included in the present systematic review.

### 3.3. Risk of Bias of Included Studies

Among the two studies included, one [16] had a low risk of bias in three or more domains, and one [15] had a high risk of bias. A detailed description of the risk of bias in each domain among the studies is reported in Table S2.

### 3.4. Study Characteristics

The main characteristics of the included studies are summarized in Table 3. All studies were retrospective. Of these, one study comes from Singapore [15], and the other one from France [16].

### 3.5. Synthesis of the Results

Among the included studies, a total of 72 patients underwent treatment for CP, and 65 of these subsequently expressed a desire to become pregnant. Ng et al. [15] retrospectively recruited and surgically treated 53 patients with CP using three different surgical techniques (Table 3). All women expressed the desire to become pregnant after treatment. A total of 18 pregnancies were achieved with natural conception (CPR: 33.96%); however, only 10 of these evolved (OPR: 18.86%), and 8 resulted in miscarriages (MR: 15.09%). The Authors do not report disaggregated data regarding fertility outcomes in relation to the different surgical technique. Nikodijevic et al. [16] retrospectively recruited 19 women with a diagnosis of CP. Twelve patients (63.16%) attempted pregnancy after undergoing treatment. Of these, 2 patients (16.67%), after having been subjected to an intramuscular injection of 1 mg/kg MTX, achieved a normally evolving pregnancy; of the 10 patients surgically treated, only 5 achieved a normally evolving pregnancy. No miscarriages were documented. The authors recorded a CPR and an OPR of 58.33% and an MR of 0.00%.

## 4. Discussion

Although CP accounts for only approximately 2% of EPs, it carries a higher risk of maternal mortality than the more common ones. Its peculiar location and the poor myometrial thickness that characterizes uterine horns can also lead to massive hemoperitoneum in the case of rupture/fissuration. The data from our study are consistent with those in the literature in terms of signs and symptoms. In fact, several authors have reported how acute pelvic pain and metrorrhagia represent pivotal symptoms [17,18,19]. The almost constant presence of pelvic pain could be explained by the fact that the uterine horn is more innervated than the rest of the fallopian tubes, so dilatation of the uterine horn would quickly lead to pelvic pain [20]. Our study also recorded three cases of hemoperitoneum (17.64%), although this finding may seem high for a relatively small patient cohort (n = 17), such as the one we recruited. This percentage is lower than the results found in the literature, which ranged from 25% to 47% [16,21,22]. The β-hCG levels we recorded are also consistent with those found by other authors. In terms of risk factors, our study did not report a high presence of these factors among the recruited patients. Only one woman in our population used IUD as a contraceptive method. IUD use increases the risk of EP, but, although it does not appear to be a specific factor in the occurrence of CP [23,24], one case of CP occurrence in a levonorgestrel-IUD-carrying woman has been reported in the literature [25]. None of the patients in our study had a history of upper genital infection. None of our patients had a history of PID. As with the IUD, a history of upper genital infection or PID is certainly a risk factor for EP, but it does not seem particularly responsible for the onset of CP [24]. Moreover, none of our patients had a history of EP. A history of EP is a risk factor for EP recurrence in general, but has no specific relationship to the onset of CP [24]. Of the 17 women studied, 16 pregnancies were spontaneous, and only 1 was an ART pregnancy. From this perspective, ART does not seem to have a statistically significant effect on the occurrence of CP [16,21]. Overall, CP can be considered a difficult condition to diagnose and treat [26]. US findings that allow a confident diagnosis of CP include an eccentrically located gestational sac surrounded by asymmetrical myometrial tissue with an empty uterine cavity [27]. Although the available literature reports excellent sensitivity and specificity for diagnosis in the presence of these US criteria, as confirmed by our study, in which they were found in only 35.30% of patients, and by the findings of other authors [28], they are not always present or easily detectable; therefore, the diagnosis is often intraoperative or, worse, may be confused with other variants of EP, such as IP [29,30,31]. It is precisely IP that is a major limitation for the studies found in the available literature. Indeed, as shown in Figure 1, 72 articles among the 88 screened to assess its eligibility in our study either confused IP with CP or associated them by understanding them as the same variant of EP [32]. However, assimilating two different types of pathologies with a natural history occurring in two distinct anatomical areas (the uterine horn and tubal isthmus) as a single variant of EP generates conceptual errors that result in the inability to reach valid diagnostic and therapeutic conclusions. Other limitations of our study are the small sample of patients in whom horn pregnancy could be ascertained by US, and the retrospective design. However, there are several strengths of our study. Despite the extreme rarity of the pathology, we were able to collect one of the largest case series in the literature, and we were able to strictly distinguish it from IP by definitive histologic examination so that there was no confounding bias. Multiple treatments were also evaluated. Finally, this study represents the first qualitative analysis aimed at defining the reproductive outcomes of CP patients undergoing the various treatments available today. To date, there is no precise management protocol in the literature because of the low incidence of CP [6]. The patients we recruited, based on the criteria established in the “Methods” section, were treated with medical or surgical therapy. In our study, medical therapy was by the US-guided injection of 1 mg/kg MTX in situ combined or in situ with an L/S-assisted injection of 1 mg/kg MTX. Both procedures were combined with an intramuscular injection of 1 mg/kg MTX; surgical therapy was performed by L/S in 76.92% of cases, and L/T in the remaining cases, and combined cornual resection associated with unilateral salpingectomy was performed. Both medical and surgical treatments resulted in the resolution of CP in 100% of cases. The studies included in this systematic review, however, show that treatment approaches other than those we adopted still lead to a 100% resolution rate, although recurrences and a worse obstetric outcome may occur in some cases [15]. After treatment, the reproductive outcomes reported in the literature are different, sometimes discordant, and not in line with each other (RCP: 33.96% vs. 58.33%; OPR: 18.86% vs. 58.33%; MR: 15.09% vs. 0.00%) [15,16]. However, it should be emphasized that these results are difficult to compare rigorously with each other and that the gap reported by the only two studies in the literature that evaluated fertility outcomes after treatment for CP could be justified, not only by the different size of the population samples considered (53 patients vs. 19 patients), but also by the variety of surgical techniques employed by Ng et al. [15] for CP resolution (Table 3). The data reported by our study further differ from the studies recruited in the systematic review in terms of the CPR (28.57%) and are slightly better than those reported by Ng et al. [15] in terms of the OPR (28.57% vs. 18.86%), while confirming those of Nikodijevic et al. [16] in terms of the MR (0.00%). As stated by some authors, surgical resection of the uterine horn would seem to worsen the possibility of achieving a subsequent pregnancy [24]. Indeed, from the data of Nikodijevic et al. [16] and our series, the medical treatment of CP would seem to ensure better reproductive outcomes (even without ART) than surgery. However, the extremely exiguous sample of patients taken for the analysis and the impossibility of analyzing disaggregated data on the different adoptable surgical techniques preclude the possibility of reaching solid conclusions. The implications of our study may be adopted for patients with rare localizations of EC, such as CP. US should guide the diagnosis of CP; however, given the difficulty encountered by several authors in making a diagnosis of CP, it should not be ruled out if US has not localized it and if a strong clinical suspicion remains. Both medical and surgical therapy are valid options in the case of CP; however, it is good to carry out appropriate counseling with all patients still desiring offspring and to inform them about the possibility of achieving pregnancy again depending on the treatment for the CP they are facing. Given the important lack of strong evidence, future research should focus on reproductive outcomes and not just surgical outcomes in the cases of CP or EP in general. These should be contextualized, not only by the intervention employed (medical or surgical), but also by patient characteristics (age, BMI, parity, AMH, etc.) and pregnancy characteristics (site, size, β -hCG levels, etc.).

## 5. Conclusions

CP is a rare form of EP that can quickly become life-threatening for the mother. Our series shows that the diagnosis of this condition can be difficult, and the presence of typical ultrasound signs may not always be detectable. In the absence of specific recommendations, the treatment of CP has been essentially surgical, based on the resection of the uterine horn combined with ipsilateral salpingectomy. In the presence of life-threatening complications, these must be managed promptly by converting to L/S or directly intervening with L/T. Medical treatment with MTX also remains an attractive and effective therapeutic alternative in selected asymptomatic patients. Our study shows that both medical and surgical treatments can safely and effectively resolve the totality of CP cases; therefore, after a rigorous study of the patient, both can be considered in the management of this scenario. To the best of our knowledge, this also represents the first systematic review on the reproductive outcomes of CP treatment. The presence of highly variable data among the recruited studies may be justified by the heterogeneity of the treatment to which patients underwent. In addition, although surgical treatment seems to be the best treatment for the purpose of achieving future pregnancy, this could be significantly influenced by the high number of patients treated surgically compared to those in whom medical treatment was used. The latter, moreover, would seem promising in terms of the CPR, OPR, and MR. In any case, the patient must be involved in the treatment decision, explaining to her the various possible treatment alternatives and their consequences. Based on these data, we take this opportunity to solicit further studies specifically designed to investigate the actual effect of medical and surgical therapy on the future fertility of patients treated for CP.

## Figures and Tables

**Table 1:** Baseline characteristics of the patients.

Patients	
Age (years), mean ± SD (range)	36 ± 4.45 (24–41)
Nulliparous, n (%)	0 (0.00)
Multiparous, n (%)	17 (100)
Previous pregnancy, mean± SD (range)	4 ± 2.04 (1–6)
Previous live birth, mean ± SD	1.31 ± 0.72
Previous miscarriage, mean ± SD	1 ± 1.05
**Pregnancy data**	
Natural pregnancy, n (%)	16 (94.12)
ART pregnancy, n (%)	1 (5.88)
Gestational age at diagnosis of CP (weeks), mean ± SD	6.85 ± 1.55
β-hCG level, IU/L, mean ± SD	15,708.06 ± 7042.06
**Risk factors for CP**	
History of EP, n (%)	0 (0.00)
Story of PID, n (%)	0 (0.00)
Infertility, n (%)	1 (5.88)
Previous abdominopelvic surgery, n (%)	0 (0.00)
Previous uterine cavity revisions, mean ± SD	1.05 ± 0.74
Presence of IUD, n (%)	1 (5.88)
Oral contraceptives, n (%)	2 (11.76)
Congenital anomalies, n (%)	0 (0.00)
Smoke, n (%)	0 (0.00)
**Signs and symptoms**	
Amenorrhea, n (%)	7 (41.18)
Metrorrhagia, n (%)	10 (58.82)
Pelvic pain, n (%)	15 (88.23)
Acute abdominal tenderness, n (%)	9 (52.94)
Palpable uterine deformation, n (%)	4 (23.53)
Systolic blood pressure under 100 mmHg, n (%)	4 (23.53)
Tachycardia, n (%)	7 (41.18)
Hemoperitoneum, n (%)	3 (17.64)
Hemorrhagic shock, n (%)	3 (17.64)

SD: standard deviation; ART: assisted reproductive technology; CP: cornual pregnancy; EP: ectopic pregnancy; PID: pelvic inflammatory disease; IUD: intrauterine device.

**Table 2:** Diagnosis and treatment.

Ultrasound Examination	
Empty uterine cavity, n (%)	17 (100)
Eccentric gestational sac surrounded asymmetrically by the myometrium, n (%)	6 (35.30)
Hemoperitoneum, n (%)	3 (17.65)
Definitive US diagnosis of CP, n (%)	6 (35.30)
**Medical treatment, n (%)**	4 (23.53)
US-guided injection of 1 mg/kg MTX in situ combined with an intramuscular injection of 1 mg/kg MTX, n (%)	1 (25)
L/S-assisted injection of 1 mg/kg MTX in situ combined with an intramuscular injection of 1 mg/kg MTX, n (%)	3 (75)
Additional procedures required	
- Second intramuscular injection of 1 mg/kg MTX, n (%)	2 (50)
Time to β-hCG negativization, days, mean	36.4
Efficacy, n (%)	4 (100)
**Surgical treatment, n (%)**	13 (76.47)
L/S–cornual resection combined with unilateral salpingectomy, n (%)	10 (76.92)
L/T conversion, n (%)	3 (30)
Intraoperative complications	
- Significant blood loss, n (%)	3 (30)
Postoperative complications	
- Severe anemia requiring transfusion, n (%)	2 (20)
L/T–cornual resection combined with unilateral salpingectomy, n (%)	3 (23.08)
Intraoperative complications, n (%)	0 (0.00)
Postoperative complications	
- Bowel obstruction, n (%)	1 (33.33)
Additional procedures required	
- Intramuscular injection of 1 mg/kg MTX, n (%)	3 (23.08)

US: ultrasound; CP: cornual pregnancy; L/S: laparoscopy; MTX: methotrexate; L/T: laparotomy.

**Table 3:** Fertility data after treatment for CP in the literature and in our series.

	Ng et al. [15]	Nikodijevic et al. [16]	Our Series
Patients undergoing treatment for CP, n	53	19	17
Patients desiring pregnancy after treatment for CP, n (%)	53 (100)	12 (63.16)	7 (41.18)
**Treatment items**			
US-guided injection of 1 mg/kg MTX in situ combined with an intramuscular injection of 1 mg/kg MTX, n (%)	0 (0.00)	0 (0.00)	1 (14.28)
Intramuscular injection of 1 mg/kg MTX, n (%)	0 (0.00)	2 (16.67)	0 (0.00)
L/S-assisted injection of 1 mg/kg MTX in situ combined with an intramuscular injection of 1 mg/kg MTX, n (%)	0 (0.00)	0 (0.00)	1 (14.28)
L/S–cornual resection combined with unilateral salpingectomy, n (%)	0 (0.00)	0 (0.00)	5 (71.43)
L/S–cornual resection, n (%)	13 (24.53)	10 (83.33)	0 (0.00)
L/S–wedge resection, n (%)	33 (62.26)	0 (0.00)	0 (0.00)
L/S–salpingectomy, n (%)	7 (13.21)	0 (0.00)	0 (0.00)
Second intramuscular injection of 1 mg/kg MTX, n (%)	9 (16.98)	0 (0.00)	0 (0.00)
**Fertility items**			
Pregnancy, n	18	7	2
Clinical pregnancy rate, (%)	33.96	58.33	28.57
Ongoing pregnancy, n	10	7	2
Ongoing pregnancy rate, %	18.86	58.33	28.57
Miscarriage, n	8	0	0
Miscarriage rate, %	15.09	0.00	0.00
Natural pregnancy, n (%)	18 (100)	7 (100)	1 (50.00)
ART pregnancy, n (%)	0 (0.00)	0 (0.00)	1 (50.00)
Pregnancy achieved after US-guided injection of 1 mg/kg MTX in situ combined with an intramuscular injection of 1 mg/kg MTX, n (%)	/	/	1 (50.00)
Pregnancy achieved after intramuscular injection of 1 mg/kg MTX, n (%)	/	2 (28.57)	/
Pregnancy achieved after L/S-assisted injection of 1 mg/kg MTX in situ combined with an intramuscular injection of 1 mg/kg MTX, n (%)	/	/	1 (50.00)
Pregnancy achieved after L/S–cornual resection combined with unilateral salpingectomy, n (%)	/	/	0 (0.00)
Pregnancy achieved after L/S–cornual resection, n (%)	NS	5 (71.42)	0 (0.00)
Pregnancy achieved after L/S–wedge resection, n (%)	NS	/	/
Pregnancy achieved after L/S–salpingectomy, n (%)	NS	/	/

CP: cornual pregnancy; US: ultrasound; MTX: methotrexate; L/S: laparoscopy; ART: assisted reproductive technology; NS: not specified.

## Data Availability

An anonymized full dataset would be available from the first author (F.M.) upon reasonable request.

## Supplementary Materials

The following supporting information can be downloaded at: https://www.mdpi.com/article/10.3390/medicina60010186/s1, Table S1: Modified Newcastle–Ottawa scoring items; Table S2: Risk of bias assessment.
