# Supplementary material for: Cornual Pregnancy: Results of a Single-Center Retrospective Experience and Systematic Review on Reproductive Outcomes
Source: Medicina (Kaunas). 2024 Jan 21;60(1):186. doi: 10.3390/medicina60010186 (PMC10819158; doi:10.3390/medicina60010186)
Supplement: Supplementary file 1 [file medicina-60-00186-s001.zip › Table S1.pdf]

**(1)**	**Study design and sample representativeness:**<br>1 point: Study design involved a control group; sample size was greater than or equal to 100 participants and exclusion rate was lower than 20%.<br>0 points: Uncontrolled study; sample size less than 100 participants or exclusion rate higher than 20%.
**(2)**	**Sampling technique:**<br>1 point: Patients recruited consecutively or randomly (randomization criteria clarified).<br>0 points: Potential convenience sampling or unspecified sampling technique.
**(3)**	**Evaluation of the description of the medical treatment and/or surgical technique used:**<br>1 point: The authors provided a comprehensive description of the medical treatment and/or surgical technique used.<br>0 points: The study does not have comprehensive description of the medical treatment and/or surgical technique used.
**(4)**	**Quality of population description:**<br>1 point: The study reports a clear description of the population (e.g., age, BMI, number of previous pregnancies, number of previous miscarriages, possible presence of previous ectopic pregnancy factors, possible presence of risk factors for the occurrence of ectopic pregnancies, etc.) with appropriate measures of dispersion (e.g., mean, standard deviation).<br>0 points: The study did not report a clear description of the population, incompletely reported descriptive statistics, or did not report dispersion measures.
**(5)**	**Incomplete outcome data:**<br>1 point: The study reported complete data on reproductive outcomes and medical and/or surgical outcomes.<br>0 points: Selective reporting of data cannot be excluded.

Table S1. Modified Newcastle-Ottawa scoring items.
The individual components listed above are summed to generate a total modified Newcastle-Ottawa risk of bias score for each study. Total scores range from 0 to 5.
For the total score grouping, studies were judged to be of low risk of bias (≥3 points) or high risk of bias (<3 points).
