# Supplementary material for: Cornual Pregnancy: Results of a Single-Center Retrospective Experience and Systematic Review on Reproductive Outcomes
Source: Medicina (Kaunas). 2024 Jan 21;60(1):186. doi: 10.3390/medicina60010186 (PMC10819158; doi:10.3390/medicina60010186)
Supplement: Supplementary file 1 [file medicina-60-00186-s001.zip › Table S2.pdf]

Author	Study design and sample representativeness	Sampling technique	Evaluation of the description of the medical treatment or surgical technique used	Quality of population description	Incomplete outcome data	Total score	Risk of bias
Ng et al. [15]	-	-	★	★	★	★★★	Low
Nikodijevic et al. [16]	-	-	★	★	-	★★	High

Table S2. Risk of bias assessment.
